# Beyond Microsatellite Instability: Intrinsic Disorder as a Potential Link Between Protein Short Tandem Repeats and Cancer

**DOI:** 10.3389/fbinf.2021.685844

**Published:** 2021-06-08

**Authors:** Max A. Verbiest, Matteo Delucchi, Tugce Bilgin Sonay, Maria Anisimova

**Affiliations:** ^1^ Institute of Applied Simulation, School of Life Sciences and Facility Management, Zürich University of Applied Sciences, Wädenswil, Switzerland; ^2^ Institute for Computational Science, Faculty of Science, University of Zurich, Zurich, Switzerland; ^3^ Swiss Institute of Bioinformatics, Lausanne, Switzerland; ^4^ Ecology, Evolution and Environmental Biology Department, Columbia University, New York City, NY, United States

**Keywords:** short tandem repeats, microsatellites, microsatellite instability, intrinsic disorder, cancer, computational biology, protein bioinformatics

## Abstract

Short tandem repeats (STRs) are abundant in genomic sequences and are known for comparatively high mutation rates; STRs therefore are thought to be a potent source of genetic diversity. In protein-coding sequences STRs primarily encode disorder-promoting amino acids and are often located in intrinsically disordered regions (IDRs). STRs are frequently studied in the scope of microsatellite instability (MSI) in cancer, with little focus on the connection between protein STRs and IDRs. We believe, however, that this relationship should be explicitly included when ascertaining STR functionality in cancer. Here we explore this notion using all canonical human proteins from SwissProt, wherein we detected 3,699 STRs. Over 80% of these consisted completely of disorder promoting amino acids. 62.1% of amino acids in STR sequences were predicted to also be in an IDR, compared to 14.2% for non-repeat sequences. Over-representation analysis showed STR-containing proteins to be primarily located in the nucleus where they perform protein- and nucleotide-binding functions and regulate gene expression. They were also enriched in cancer-related signaling pathways. Furthermore, we found enrichments of STR-containing proteins among those correlated with patient survival for cancers derived from eight different anatomical sites. Intriguingly, several of these cancer types are not known to have a MSI-high (MSI-H) phenotype, suggesting that protein STRs play a role in cancer pathology in non MSI-H settings. Their intrinsic link with IDRs could therefore be an attractive topic of future research to further explore the role of STRs and IDRs in cancer. We speculate that our observations may be linked to the known dosage-sensitivity of disordered proteins, which could hint at a concentration-dependent gain-of-function mechanism in cancer for proteins containing STRs and IDRs.

## 1 Introduction

Short Tandem Repeats (STRs), also known as microsatellites, are genomic motifs of 1–6 base pairs that are repeated back-to-back. STRs are estimated to make up around 3% of the complete human genome (Ellegren, 2004). They are highly polymorphic, with a mutation rate that is estimated to be several orders of magnitude higher than non-repeating sequence (Willems et al., 2014). The primary mode of mutation in STRs is their contraction or expansion by gain or loss of repeat units. The process that is mainly held responsible for this is replication slippage (Viguera et al., 2001). In this process, one of the two DNA strands ’slips’ during replication, forming a hairpin-like structure. Depending on which of the two strands slips, this can lead to either insertions or deletions of repeat units.

STRs occur most often in non-translated parts of the genome (Ellegren, 2004). In promoter regions, they were found to affect gene expression divergence between human and several great ape species (Bilgin Sonay et al., 2015a). This effect was more pronounced for STRs with shorter repeat units and those occurring closer to the transcription start sites of genes. STRs have been shown to exert their gene regulatory effects through a variety of mechanisms, including alteration of transcription factor binding affinities (Martin et al., 2005), histone modification (Gymrek et al., 2016) and DNA methylation (Quilez et al., 2016). While the majority of STRs are found in non-coding regions, they appear in protein-coding regions of the genome as well (Delucchi et al., 2020). In proteins, STRs are strongly enriched with disorder promoting amino acids, and are primarily found in intrinsically disordered regions (IDRs) (Jorda et al., 2010; Delucchi et al., 2020). IDRs are parts of proteins that do not settle into a fixed secondary or tertiary structure, and instead remain unfolded in isolation (Uversky, 2013). Through their flexibility, IDRs confer the ability to bind and interact with a wide variety of target proteins (Babu et al., 2012; Uversky, 2013). The fraction of proteins with IDRs increases with organism complexity, due to an evolutionary process hypothesized to be driven by expansions of STRs located in IDRs (Tompa, 2003).

Apart from their physiological functions, both STRs and IDRs are implicated in cancer (Iakoucheva et al., 2002; Hause et al., 2016). Colorectal cancer (CRC) tumors, for example, were found to be enriched with STR unit number variations in promoter and exonic regions (Bilgin Sonay et al., 2015b). This is especially true for tumors with a microsatellite instability-high (MSI-H) phenotype. These tumors typically display defects in the DNA mismatch repair (MMR) system, which allows for the frequent contraction and expansion of STRs (Evrard et al., 2019). MSI is the most prevalent for endometrial, gastric and colorectal cancers, where around 30, 20 and 15% of patients are classified as MSI-H, respectively (Guinney et al., 2015; Hause et al., 2016; Bonneville et al., 2017). In most other cancer types, the MSI-H phenotype is only sporadically observed.

While ample evidence exists linking STRs, IDRs and disease (see Darling and Uversky (2017) for a review), investigations in the context of cancer often focus on either tandem repeats or intrinsic disorder separately, disregarding their inherent association. Here we aim to demonstrate that investigating these features together can lead to a deeper understanding of their roles in cancer. To this end, we mapped both of these phenomena in all reviewed canonical human proteins from the UniProtKB/SwissProt database (The UniProt Consortium, 2019). In line with previous findings, we determined the vast majority of STRs to be made up completely of disorder-promoting amino acids. The subset of STRs consisting of ordered amino acids appear primarily in signal peptides and are cleaved from the mature protein during preprocessing. Through functional analysis of STR-containing proteins we confirmed that they are over-enriched for protein and nucleotide binding functions, and contain a larger amount of IDRs than non-STR-containing proteins. Moreover, they were enriched in various functional pathways that are associated to carcinogenesis. For several cancer types, we found STR-containing proteins to be enriched among those whose expression correlates with patient survival according to the Protein Atlas Pathology Atlas (Uhlen et al., 2017). The fact that this was observed also for cancers that display low incidence of MSI-H (e.g., pancreatic, skin and liver cancers) suggests a role for STR- and IDR-containing proteins in carcinogenesis for microsatellite stable (MSS) phenotypes as well. We postulate that the interaction promiscuity hypothesis put forward by Vavouri et al. (2009) could provide a general gain-of-function mechanism for over-expressed STR- and IDR-containing proteins in the context of cancer.

## 2 Materials and Methods

### 2.1 Data

All canonical human proteins from the UniProtKB/Swiss-Prot reviewed section of the UniProt knowledgebase (queried on December 21, 2020) were downloaded (The UniProt Consortium, 2019). This resulted in a dataset of 20,394 proteins, which was used for the analyses described in this report. All analyses described in this work were implemented in *Python* and R. Scripts and generated data sets can be found at https://github.com/acg-team/swissprot_human_strs.

### 2.2 Short Tandem Repeat Detection

STRs were detected, validated and filtered using the Tandem Repeat Annotation Library (TRAL) version 2.0 (Schaper et al., 2015). TRAL enables the integration of the output of multiple tandem repeat detection algorithms, and can be found at https://github.com/acg-team/tral. For the experiments described here, the detection algorithms were HHrepID (Biegert and Söding, 2008), T-REKS (Jorda and Kajava, 2009), TRUST (Szklarczyk and Heringa, 2004) and XSTREAM (Newman and Cooper, 2007). Tandem repeats with repeat unit length longer than two amino acids (AA) were removed in order to adhere to the definition of a STR as a repeating motif with unit length 1–6 base pairs. Using TRAL, the remaining STRs were scored based on a phylogenetic model of STR evolution. Here, a likelihood ratio test is applied to determine whether the repeat units are independent of each other, or if they arose from duplication events (i.e., they are evolutionary related) (Schaper et al., 2012). STRs that could have been formed by chance (likelihood ratio test *p*-value > 0.05) were removed, and the remaining set of validated STRs was refined to remove redundancies using common-ancestry clustering (in case of overlap, the STR with the lowest *p*-value and divergence was retained). Following this process, circular-profile hidden Markov models (cpHMM) were constructed from each repeat which were used to further refine the STRs (Schaper et al., 2014).

### 2.3 Intrinsic Disorder Prediction

IDR prediction from sequence was done using MobiDB-lite with the docker image available at https://github.com/BioComputingUP/MobiDB-lite_docker (Necci et al., 2020). Similarly to TRAL, MobiDB-lite is a consensus method that integrates the output of a variety of disorder prediction algorithms, and is optimized for the detection of long disordered regions with high accuracy. MobiDB-lite was run with default parameters, and the following disorder prediction algorithms were used: ESpritz (NMR, DisProt & X-ray) (Walsh et al., 2012), GlobProt (Linding et al., 2003b), DisEMBL (465 & hot loops) (Linding et al., 2003a) and IUpred (long & short) (Dosztanyi et al., 2005).

### 2.4 Functional Analysis of Short Tandem Repeat-Containing Proteins

Functional over-representation analysis was performed on STR-containing proteins using the g:Profiler webserver (Raudvere et al., 2019). The over-representation analysis was performed against a custom background consisting of all SwissProt proteins investigated in this report. The included data sources were the biological process, cellular component and molecular function domains from the Gene Ontology, as well as KEGG pathways. Enrichments with a Benjamini-Hochberg corrected *p*-value <= 0.05 were considered significant.

### 2.5 Short Tandem Repeat-Containing Proteins and Patient Survival in Cancer

Correlations between gene expression levels and patient survival were obtained for the 17 cancer types included in the protein atlas pathology atlas (PA) (Uhlen et al., 2017). This yielded three groups of proteins per cancer type based on their relationship to patient survival: an uncorrelated, a favourably correlated and an unfavourably correlated PA group. Combining this information with our data set, for each PA group we determined which survival-associated proteins harbored an STR. The number of STR-containing proteins in each PA group was tested for enrichment compared to the two other groups of the same cancer type using Fisher’s exact tests. A Benjamini-Hochberg correction with α=0.05 was used to control the false discovery rate.

Potential patterns of biological functions shared between STR-containing proteins in different PA groups were explored using GO terms from the biological process, molecular function and cellular component domains. To this end, GO annotations for human proteins were retrieved from http://current.geneontology.org/products/pages/downloads.html on January 26, 2021. For all PA groups, the number of STR-containing proteins mapping to each GO term was determined. GO terms that were observed in fewer than 5 PA groups were filtered out, after which we removed two PA groups that mapped to very few GO terms (prostate favourable and testis favourable, mapping to 9 and 0 terms, respectively). This filtered set of GO term counts per PA group was used as input for UMAP (McInnes et al., 2018) to create a 10-dimensional embedding, on which k-means clustering was performed. The optimal number of cluster centers was determined based on the within-cluster sum of squares (WSS).

## 3 Results

### 3.1 Length and Disorder Propensities of Human Protein Short Tandem Repeats

**FIGURE 1:**
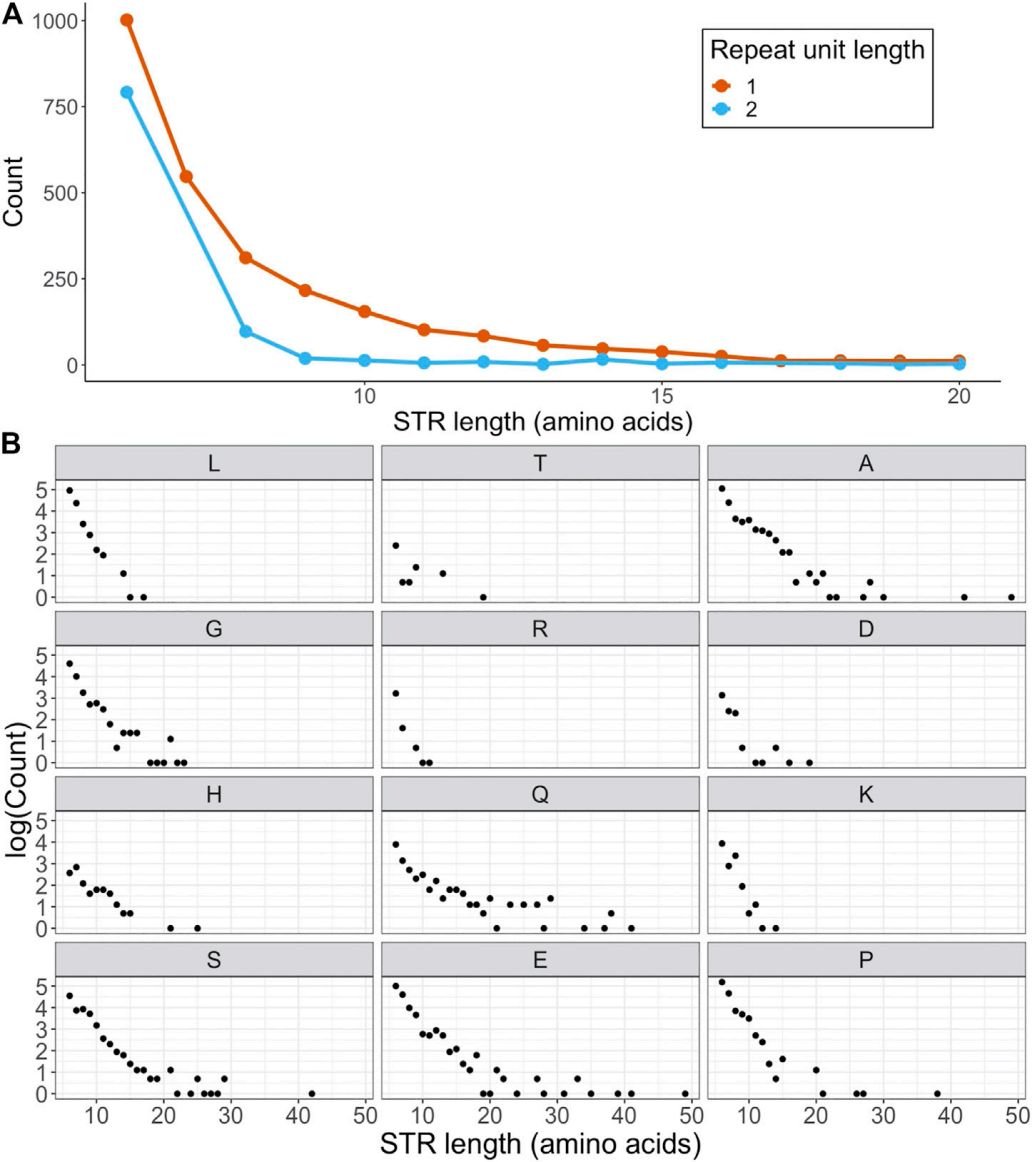
Frequencies and numbers of STRs in human SwissProt proteins. **(A)** Frequencies of STRs as a function of STR length, separated on STR unit length (STRs with length < 20 are shown). **(B)** log (counts) of different homorepeats as a function of STR length. Separated by amino acid and ordered by ascending disorder propensity. Only homorepeat types for which more than 10 cases were found are shown.

Out of the 20,394 canonical human protein sequences in SwissProt, 2,658 (13.0%) were found to be STR containing. As some proteins had more than one repeat region, the total number of STRs was 3,699. Out of the STR-containing proteins, 85.5% also contained at least one IDR - although not necessarily overlapping the STR. This was substantially lower for non-STR proteins, where 50.8% of proteins were predicted to contain an IDR. 2,717 of all STRs were homorepeats consisting of repeating tracts of a single amino acid (AA). While some of these homorepeats were over 100 AAs long, most ranged between a length of 6 and 20, with an average of 9.00 (Figure 1A). We detected fewer STRs with unit length 2, and these were generally shorter than homorepeats with an average length of 6.98 AAs (Figure 1A).

Not all AAs were equally represented in the set of homorepeats (Figure 1B). With 458 occurrences, polyA was the most common homorepeat, closely followed by polyP and polyE, each observed 453 times. The set of homorepeats was dominated by disorder promoting AAs (A, G, R, D, H, Q, K, S, E & P) (Campen et al., 2008), which was in line with previous findings (Delucchi et al., 2020). In fact, out of the 10 AAs defined as order promoting by the TOP-IDP scale (W, F, Y, I, M, L, V, N, C & T) (Campen et al., 2008), only two - leucine and threonine - had more than 10 homorepeats in human SwissProt proteins. Not a single homorepeat was made up of the order promoting amino acids isoleucine, phenylalanine and tryptophan. The only exception to this rule was leucine, which made up 290 STRs even though it is an order promoting residue.

For the 982 STRs with unit length 2 we detected 177 different combinations of AAs that constituted a repeat unit. Most of these were very infrequent, with only 29 combinations being observed in STRs more than 10 times across all human SwissProt proteins. The number of combinations was reduced to 109 if only the AAs were considered and not their order (e.g., AC equals CA). We observed that as some AA pairs showed a strong preference for appearing in one order over the other. For example, we detected 62 STRs where ’SG’ was the dominant unit, compared to 14 cases of ’GS’, and 44 times ’RS’ vs. 24 times ’SR’. To determine if these skews were specific to STRs, we also computed the background frequencies of the 8 AA combinations that appeared more than 30 times in STRs across all non-repeat sequence in human SwissProt proteins. The investigated AA combinations were ‘A-G’, ‘A-P’, ‘D-E’, ‘G-P’, ‘G-R’, ‘G-S’, ‘P-S’ and ‘R-S’. When comparing their dipeptide counts to those in STRs, we found that only the distribution of SG-GS was significantly different from the non-repeat background, although it is possible that a larger STR sample size would lead to more significant findings (Supplementary Figure S1). Thus it appears that in most cases the order of AA appearance in dipeptide STRs is not different from that observed in protein sequences in general. In the case of the exception SG-GS, we found that 35 out of 40 members of the immunoglobulin kappa variable chain family of proteins in SwissProt have an SG repeat, while none have a GS repeat. Furthermore, the Activin, BMP and TGF-*β* type-I receptors also all contain an SG repeat. This could point to a shared evolutionary background or a specific functional requirement for an SG repeat in these proteins, although further investigations are needed to elucidate this. Another point worth noting is that our investigations were performed on amino acid sequences only. It is possible that an STR appears as an ’SG’ repeat on the protein level, but is detected as a ’GS’ repeat in the DNA, for example due to the first serine being encoded by an alternate codon than the following ones. Future studies into “purity” of protein-coding STRs on the DNA level could shed light on whether this is the case or not.

**FIGURE 2:**
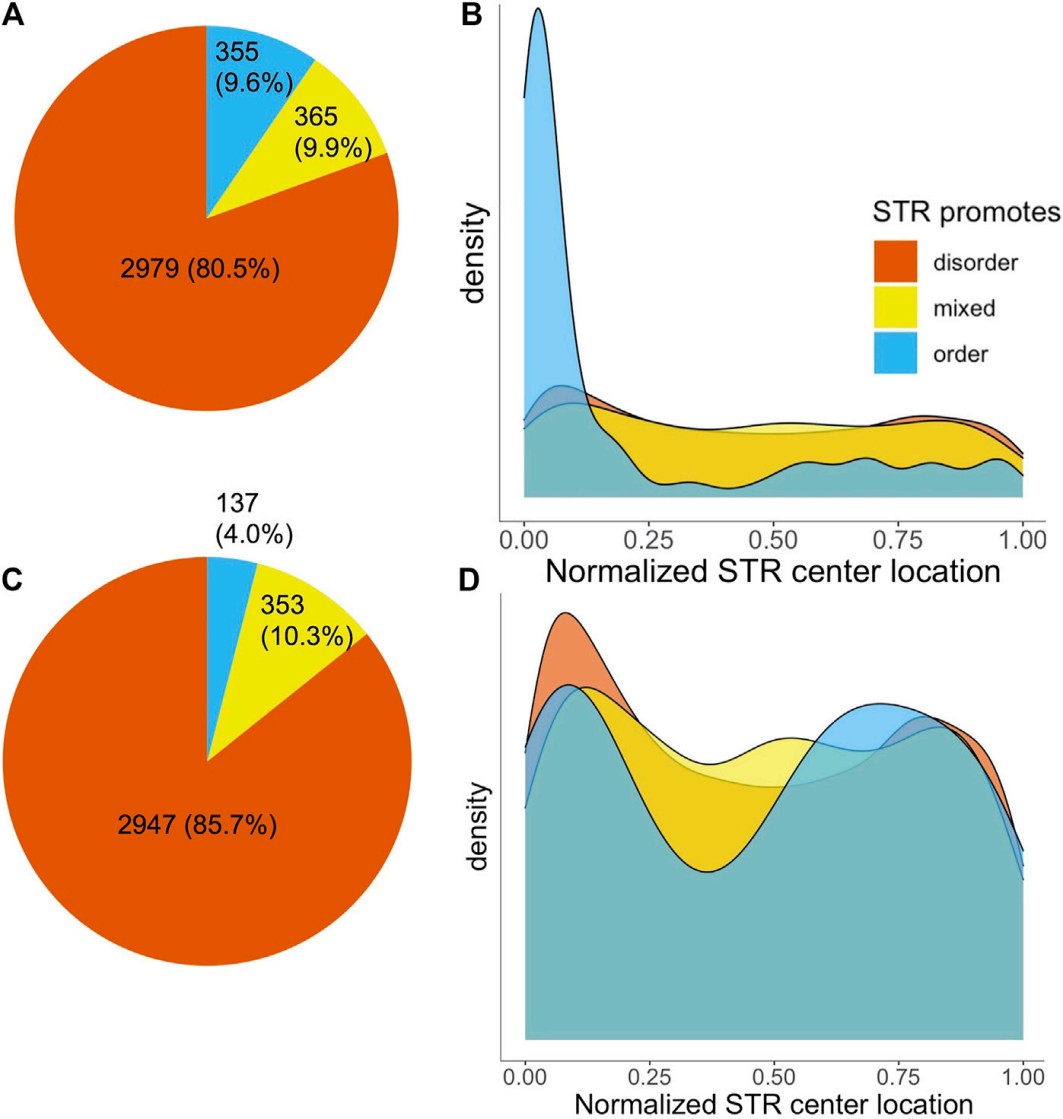
Number and localization of STRs in SwissProt proteins by disorder propensity. **(A)** Pie chart of the number of SwissProt human STRs, colored on disorder propensity. **(B)** Density plot of normalized STR center locations, split by disorder propensity. **(C,D)** The same as A and B, but after removal of STRs located in signal peptides.

The majority of STRs (2,979) consisted completely of disorder promoting amino acids. 365 STRs were made up of a mixture of order and disorder promoting amino acids, with a further 355 being fully order promoting (Figure 2A). Given the fact that STRs consist mainly of disorder-promoting amino acids it is to be expected that they are mainly found in disordered protein regions. This was confirmed by MobiDB-lite disorder predictions, as out of all amino acids in STR sequences, 62.1% were also in a predicted IDR. This was substantially higher compared to non-STR sequence, where only 14.2% of amino acids were predicted to be in an IDR. Unsurprisingly, the percentage of STR overlap with IDRs varied with the disorder propensity of the AAs making up repeat regions: the percentage of amino acids predicted as disordered was 71.2, 45.0 and 4.38% for disorder promoting, mixed and order promoting STRs, respectively.

We investigated the location of STRs in proteins by determining the position of the middle of STRs while correcting for repeat length as described in Delucchi et al. (2020). This analysis yielded a striking difference based on intrinsic disorder propensity of the amino acids in STRs. Figure 2B shows the normalized location of STR centers in proteins. The disorder promoting and mixed STRs appeared to have a slight preference for the protein termini, but otherwise were relatively evenly spread across the protein sequences. Order promoting STRs, on the other hand, occurred almost exclusively at the N-terminus of proteins. The average center location was significantly different for order promoting STRs compared to either disordered or mixed STRs (Welch’s *t*-test, p≪0.001 in both cases). We suspected this to be due to the fact that N-terminal signal peptides contain a stretch of hydrophobic order promoting amino acids (Von Heijne, 1990). This was investigated by annotating the STR containing protein sequences with information of where signal peptides occur, obtained from SwissProt. Out of the 355 ordered STRs 61.7% were located in a signal peptide, whereas only 1.1% of disordered and 3.3% of mixed STRs were in a signal peptide (Figure 2C). 215 of the 290 order promoting polyL homorepeats described in the previous section were found to be located in signal peptides. When the center locations of STRs were investigated while disregarding STRs occurring in signal peptides, the large N-terminal peak of ordered STRs was gone and average center locations no longer differed significantly between groups (Figure 2D). Signal peptides are cleaved off during preprocessing and thus not present in the mature protein. However, disruptions of signal peptides, e.g., by contraction or expansion of a STR, may have an impact on the accurate localization of proteins in the cell. An example of a signal peptide alteration having a pathogenic effect has been described for autosomal dominant familial isolated hypoparathyroidism. In this disease a Cys $\begin{array}{c} \to \end{array}$ Arg mutation disrupts the hydrophobic core of the signaling peptide of the immature parathyroid hormone protein, thereby impairing its efficient secretion (Arnold et al., 1990). Thus, we believe that signal peptide STRs also have the potential to have a biological - or even pathological - effect when they expand or contract. We therefore decided to include signal peptide STRs in our investigations and perform our downstream functional and cancer-related analyses on all 3,699 STRs.

### 3.2 Functional Analysis of Short Tandem Repeat-Containing Proteins

**FIGURE 3:**
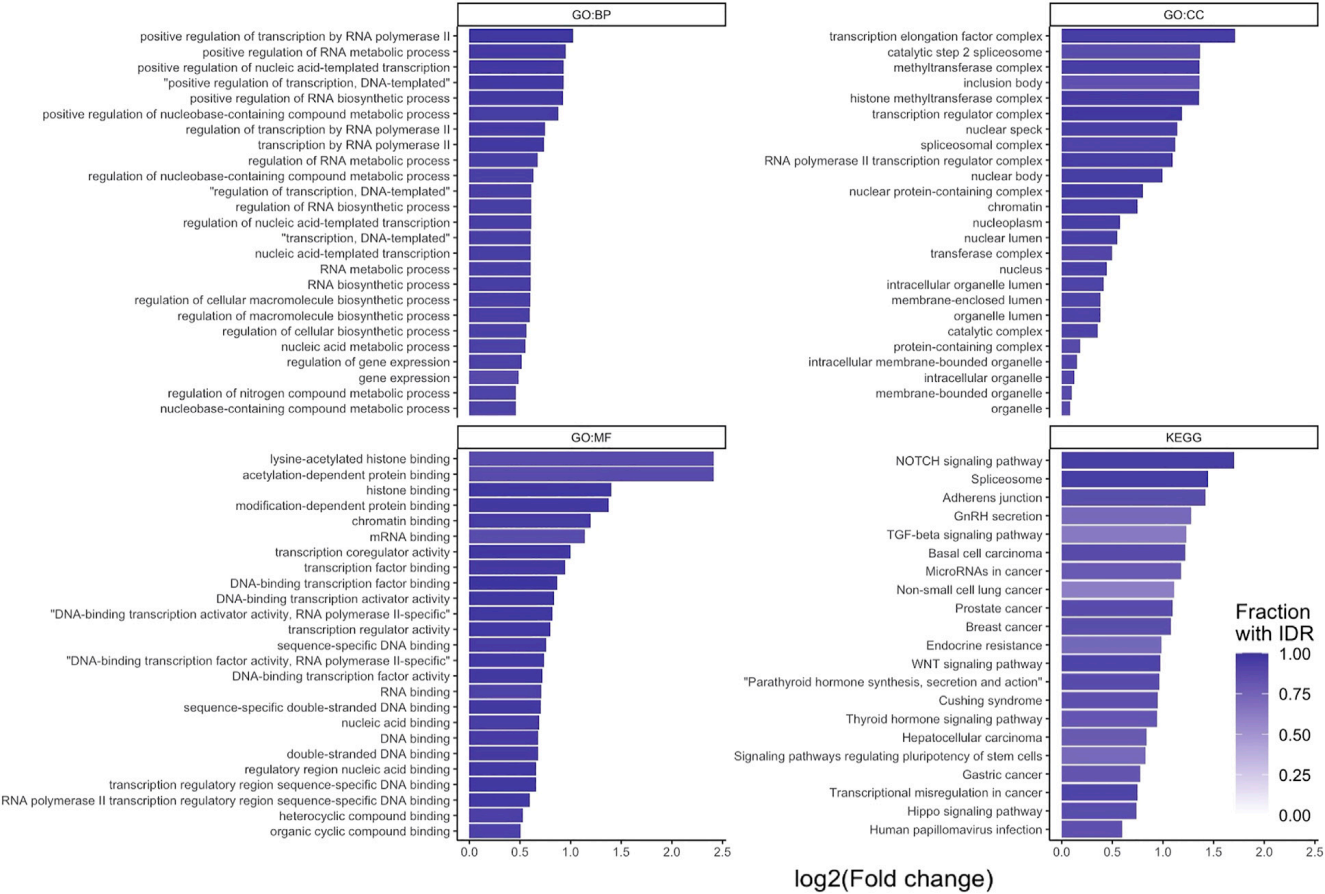
g:Profiler over-representation analysis of STR-containing proteins. log2 (fold enrichment) of significantly over-represented terms for the three gene ontology domains: biological process (GO:BP), cellular component (GO:CC) and molecular function (GO:MF), and KEGG PATHWAYS. For each data source, the 25 over-represented terms with the lowest *p*-value are shown (all 21 for KEGG). Bars are shaded based on the fraction of proteins per term that contained an IDR.

An over-representation analysis was performed on the 2658 STR containing proteins. This analysis was performed for the biological process (BP), molecular function (MF) and cellular component (CC) domains of the gene ontology, as well as KEGG pathways. This resulted in 449, 54, 87 and 21 significantly over-enriched terms, respectively (Figure 3). A full list of enriched terms can be found in the Supplementary Materials.

From the over-represented terms, it is evident that STR containing proteins are primarily found in the nucleus of cells (e.g., GO:CC ‘nucleus’, ‘nuclear lumen’, ‘chromatin’) and have a predisposition to perform binding and transcription regulatory functions (e.g., GO:MF ‘DNA binding’, ‘RNA binding’, ‘histone binding’, ‘transcription regulator activity’). Furthermore, they are involved in processes relating to gene expression and epigenetic regulation (e.g., GO:BP ‘gene expression’, ‘chromatin organization’, ‘histone modification’). As noted earlier, a large proportion of STR-containing proteins also has IDRs (Figure 3). These findings are in line with the prevailing view that disordered regions and proteins are important for protein-protein and protein-nucleotide binding functions (Dyson and Wright, 2005; Babu et al., 2012).

At the KEGG pathway level, STR-containing proteins were found to be over-represented in basal cell, prostate, breast, hepatocellular and gastric cancer associated pathways. Additionally, Notch, Wnt, TGF-*β*, and Hippo signaling pathways were over-represented. These cascades are often dysregulated in a variety of cancer types (Sanchez-Vega et al., 2018). The TGF-*β*, Wnt and Notch signaling pathways were also over-represented terms in the GO:BP domain. In the GO:MF domain, SMAD-binding (part of TGF-*β* signaling), beta-catenin binding (part of Wnt signaling) and p53 binding (a crucial regulator of the cell-cycle) were over-represented. Overall, this over-representation analysis analysis confirmed STR-containing proteins to be interesting targets in the context of cancer.

**FIGURE 4:**
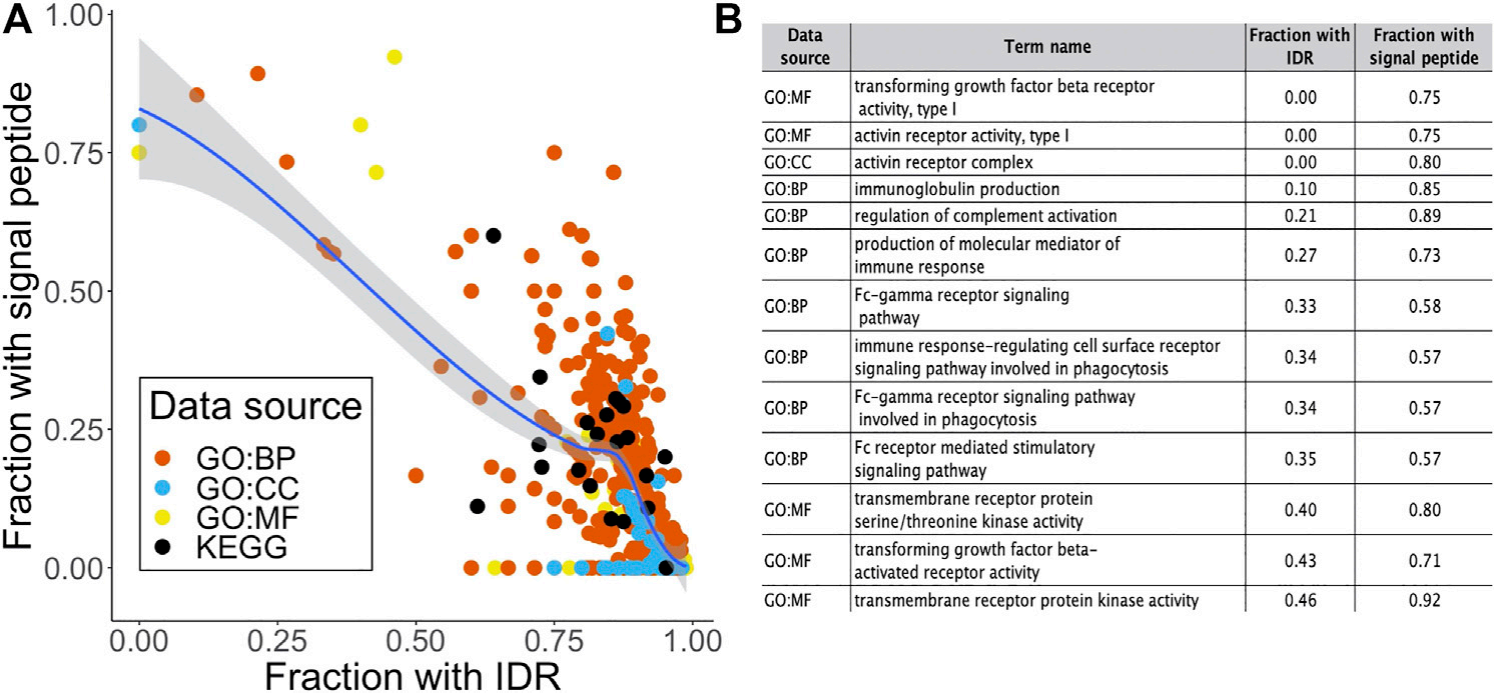
Relating signal peptide content to intrinsic disorder and biological function. **(A)** Scatter plot of the fraction of proteins in an over-represented term that contained a signal peptide as a function of the fraction of IDR-containing proteins. Dots are colored by data source. **(B)** Term name, data source and fraction of signal peptide proteins for the 15 terms with a fraction of IDR-containing proteins < 0.5.

Previously in this report, we found that 506 out of the 2658 STR-containing proteins were annotated with a signal peptide that targets proteins for secretion or membrane localization. While they were not among the most enriched terms shown in Figure 3, we could detect several significant terms relating to receptor activity in the over-representation analysis. As expected, a large proportion of proteins in these terms were annotated with a signal peptide in SwissProt. Interestingly, we found an inverse correlation between the fraction of signal peptide- and IDR-containing proteins in over-represented terms (Figure 4A). This may be linked to our previous observation that out of the 262 STR we found in signal peptides, 218 were completely order-promoting. Most enriched terms that contained a low fraction of IDRs and a high fraction of signal-peptides (top left in Figure 4A) were not related to binding or gene regulation functions. Instead, they carried out immune functions and receptor activities, and were located in membrane structures (Figure 4B). Taken together, a picture emerges of two classes of STR-containing proteins. The largest of these harbors disorder-promoting STRs and is important for gene expression and binding functions in the nucleus and cytosol, the other is made up of the subset of signal peptide-containing proteins that are translocated to the cell surface to carry out their receptor functions. This hypothesis was further tested by running GO over-representation analyses for the set of signal peptide containing STR proteins and disorder promoting STR proteins (without a signal peptide) separately. For the signal peptide STR proteins, this yielded 503, 83 and 105 over-represented terms for the GO BP, CC and MF domains, respectively. The most enriched terms were overwhelmingly related to extracellular and membrane compartments, and described cell adhesion and receptor processes and functions (Supplementary Figure S2A). On average, 55.0% of signal peptide STR proteins per GO term were also predicted to contain an IDR. This was substantially lower than the 86.4% that we found when looking at all STR-containing proteins together. For the subset of disorder promoting STR proteins without signal peptides, the findings were quite similar to the analysis on the full set of STR-containing proteins: enriched terms were related to the cellular compartment and binding and gene regulatory functions (Supplementary Figure S2B). For these terms, the average percentage of proteins predicted to have an IDR was even higher than found in the analysis of all STR-containing proteins: 90.1%.

### 3.3 Short Tandem Repeats in Cancer-Related Proteins

Following up on our findings that STR-containing proteins were enriched with cancer-related functions and pathways, we investigated the occurrence of STRs in the protein atlas pathology atlas (PA) (Uhlen et al., 2017). In the PA, gene expression levels at time of diagnosis are correlated to patient survival for 17 cancer types from different anatomical sites. For example, the following links to the PA show Kaplan-Meier plots of a favourable and unfavourable STR-containing protein in colorectal cancer: DNA mismatch repair protein Msh3 https://www.proteinatlas.org/ENSG00000113318-MSH3/pathology/colorectal+cancer, Regulator of cell cycle RGCC https://www.proteinatlas.org/ENSG00000102760-RGCC/pathology/colorectal+cancer. The information in the PA was merged with our set of STR-containing proteins, allowing for an investigation of STR occurrence in cancer-associated proteins (see Methods).

For every anatomical site we determined how many STR-containing proteins were in the uncorrelated, favourably correlated or unfavourably correlated group of proteins according to the PA. In total, 1499 STR-containing proteins were present in at least one PA group. The majority of these occurred in only one or two PA groups. The most shared protein was Integrin alpha-5, which correlated with unfavourable patient survival in six cancer types, and favourable survival in one. In general, the different PA groups were found to harbor distinct STR-containing proteins, with few proteins shared between more than two cancers.

**FIGURE 5:**
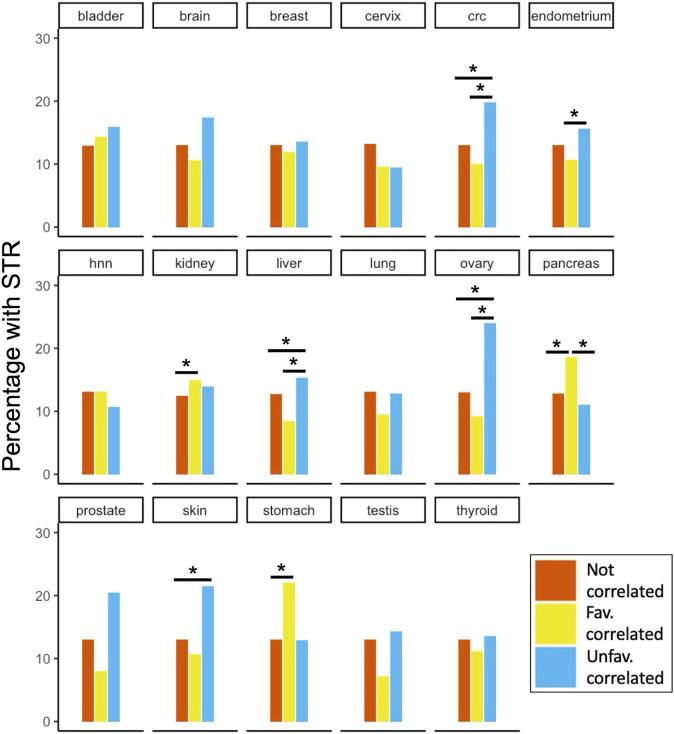
STR-containing proteins and their correlation to patient survival in cancer. For all 17 cancer types included in the Protein Atlas Pathology Atlas, the percentage of STR-containing proteins among the proteins that were either uncorrelated or correlated (un-)favourably with patient survival are shown. Significant enrichments after Benjamini-Hochberg FDR correction are marked with asterisks. Note that while percentages are shown in the plot for clarity, tests were performed on absolute numbers of proteins. Abbreviations: hnn, head and neck; crc, colorectal cancer.

The STR content in each PA group was tested for enrichment compared to the two other groups for each site (Figure 5). We found significantly enriched groups in colorectal cancer (CRC), endometrial, renal, hepatic, ovarian, pancreatic, skin and gastric cancers. For renal, gastric and pancreatic cancers, the favourably correlating proteins contained significantly more STRs compared to one or both other groups. For colorectal, endometrial, hepatic, ovarian and skin cancers, on the other hand, the unfavourably correlating proteins were enriched with STRs. It is noteworthy that for some of the other cancer types (e.g., prostate and testis) there was quite a large difference in the fraction of STR-containing proteins between the PA groups shown in Figure 5, but no significant enrichment. The reason for this was likely due to the small number of survival correlated STR-containing proteins for these cancer types compared to others (see https://www.proteinatlas.org/humanproteome/pathology for an overview). The analysis of STR enrichment among cancer-associated proteins was repeated for the subset of disorder promoting STRs (Supplementary Figure S3). This yielded very similar results to those shown in Figure 5, and the overall trends observed for the different cancer types were preserved. However, due to the smaller number of STR-containing proteins in this subset, some enrichments of PA groups were no longer significant. Specifically, colorectal cancer unfavourable, renal cancer favourable and gastric cancer favourable PA favourable groups were no longer significantly enriched with STR-containing proteins when only considering disorder promoting STRs.

**FIGURE 6:**
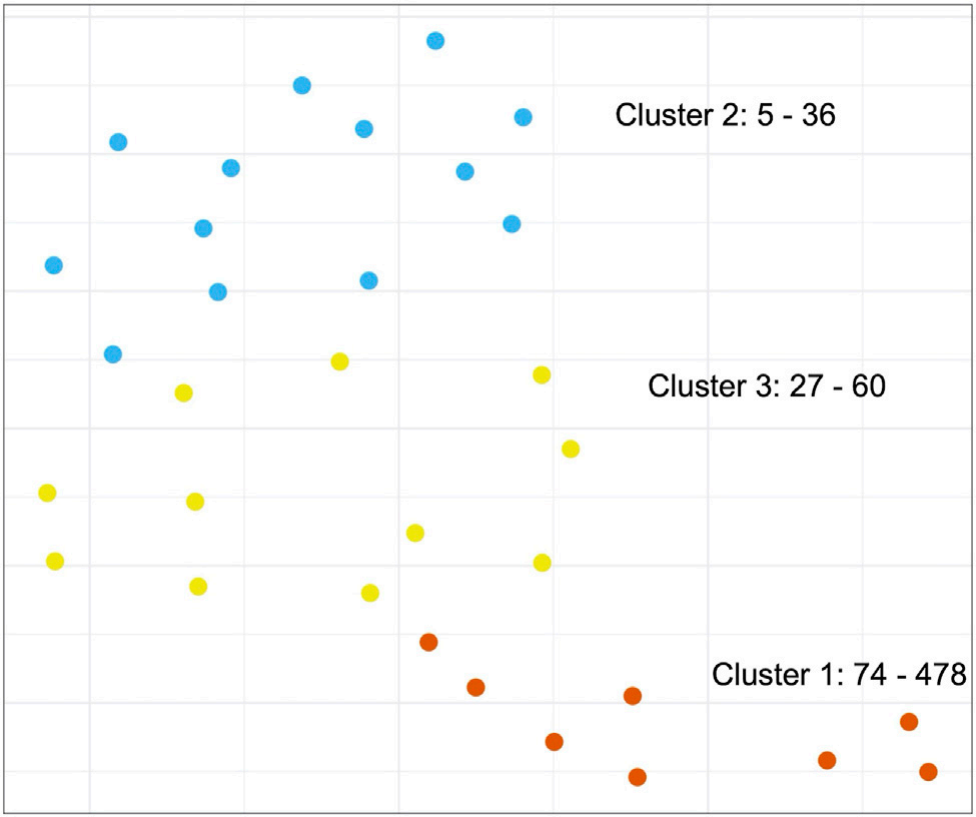
Clustering of biological functions of STR-containing proteins in the Protein Atlas Pathology Atlas. K-means clustering of the UMAP-embedded number of STR-containing proteins associated to Gene Ontology terms for all PA groups. Text annotations in the graph refer to the PA groups in each cluster with the smallest and largest number of STR-containing proteins. Note: while clustering was performed on a 10-dimensional embedding, the results are visualized here using a 2-dimensional embedding.

Next, we set out to investigate whether we could detect similarities between cancer types based on patterns of STR content across PA groups. Since we already observed that STR-containing proteins were generally not shared between PA groups, we instead decided to focus on GO terms for this analysis in order to discern patterns at a higher, functional level (see Methods). Using UMAP (McInnes et al., 2018), an embedding of the number of STR-containing proteins mapping to GO terms for each PA group was generated. This was used as input for k-means clustering (Figure 6). While we did observe three separated clusters, it appeared that the clustering was based mostly on the number of STR-containing proteins per PA group, rather than any biological reason. To address this, the procedure was repeated using log transformed count data, however this did not change the findings much (Supplementary Figure S4A). In a final attempt, we scaled the GO term counts for each PA group by dividing the count values by the number of STR-containing proteins found for that group. After embedding and clustering, the PA groups no longer appeared to be grouped based on the number of STR-containing proteins. However, the resulting plot showed the PA groups scattered quite evenly across the embedding space, with no obvious, separated clusters (Supplementary Figure S4B). From this we concluded that there were no shared patterns of GO terms between the PA groups of different cancer types, and that any patterns that we did observe were based on the PA group size rather than biology.

While we could not detect patterns of biological functions across the different cancer types, the fact that we found enrichments in several cancers allows for interesting inferences about the role of STR-containing proteins. In the context of cancer, STRs are typically studied through the scope of microsatellite instability (MSI). MSI-H is a phenotype observed in several cancer types where many alterations in STRs are observed in tumors due to defects in the DNA mismatch repair (MMR) system. It is the most prevalent in endometrial (30% of cases), gastric (20%) and colorectal cancers (15%) (Hause et al., 2016; Bonneville et al., 2017). While we did find enrichments in one of the PA groups for each of these cancer types, we also observed enrichments for cancers that have a very low proportion of MSI-H tumors. Investigations of skin, ovarian, pancreatic, liver and kidney cancers report between 0 and 3% of tumors with MSI-H phenotype (Hause et al., 2016; Bonneville et al., 2017). The fact that we could detect enrichments of STR content in proteins that correlate with patient survival for these cancer types suggests that the role protein STRs play in cancer is not limited to MSI-H tumors. Another interesting observation was that STR enrichments were not limited to either favourable or unfavourable PA groups. This indicates that STR-containing proteins are not universally positive or negative for patient survival in cancer, but that their role is context dependant. A possible reason for this may be found in the interaction promiscuity hypothesis that Vavouri et al. (2009) put forward to explain why certain genes are harmful when over-expressed, but others are not. In their study, both IDRs and linear motifs were identified as important determinants of dosage-sensitivity. IDRs and the STRs often contained therein tend to have many off-target, low affinity binding partners (Babu et al., 2011). When the concentration of these proteins increases, more of these off-target binding instances will actually occur in the cell due to mass-action kinetics. This can in turn alter interaction networks and modulate cellular behavior. Because the PA contains correlations of gene expression levels to patient survival, it is possible that it preferentially identifies dosage-sensitive proteins. According to Vavouri et al. (2009), these proteins should be enriched with both STRs and IDRs, which is what we observe here for eight cancer types from the PA.

In this light, over-expression of STR- and IDR-containing proteins could provide a general gain-of-function mechanism by increasing the likelihood of off-target binding and interactions. The effect of such an expanded set of binding partners is difficult to predict, which is reflected in the fact that STR-containing proteins are enriched in both favourable and unfavourable PA groups for different cancers. Future studies should be conducted to elucidate the dosage-dependent binding capabilities of STR-containing proteins in the context of cancer. Such studies should preferentially be focused on the cancer types for which we found enrichments of STR content in the PA groups, i.e. colorectal, endometrial, renal, hepatic, ovarian, pancreatic, skin and gastric cancers. It will be of particular interest to stratify cancers into subtypes to investigate STR-containing proteins across different phenotypes. It is possible that the type of data in the PA, which was derived without stratification of cancer subtypes, masks a more diverse pattern within each cancer with potentially more pronounced enrichments than we observed here.

## 4 Discussion

In this report, we explored the occurrence of short tandem repeats and intrinsic disorder in a non-redundant set of human proteins spanning the proteome. We could confirm previous findings that STRs are made up primarily of disorder-promoting amino acids, and are much more likely to be predicted to occur in disordered regions than non-repeating protein sequence. Interestingly, this was reversed in signal peptides where many - mostly polyL - ordered homorepeats were observed. Functional analysis of STR containing proteins showed that they are preferentially located in the nucleus of cells, where they are important for the regulation of gene expression and have protein-protein and protein-nucleotide binding functions. Here we could also subdivide the group of STR-containing proteins into two categories on the basis of the presence of signal peptides. Signal peptide containing STR proteins were found to be located in the cell membrane and extracellular matrix, where they performed primarily receptor-related functions. They were less likely to be predicted to contain IDRs. As we could also find many enrichments in cancer-related signaling pathways, we mapped our annotations of protein STRs to genes whose expression is known to correlate with patient survival in cancer (Uhlen et al., 2017). Through this analysis, we could show that STR content was enriched in both favourably and unfavourably correlating proteins for cancers originating from eight different anatomical sites. When looking at only proteins with disorder promoting STRs, three of these cancer types no longer showed significant enrichments, even though the overall trends were preserved. These findings could potentially stem from the fact that IDR-containing proteins typically have many low affinity binding partners (Uversky, 2013). As the concentrations of these proteins increase by higher gene expression, mass-action kinetics will cause more of these low-affinity interactions to take place (Vavouri et al., 2009). This could constitute a concentration-dependent gain-of-function mechanism for STR- and IDR-containing proteins. Future research should aim to determine to what extent this mechanism is involved in cancer.

While it has long been known that STRs and IDRs are tightly linked and often co-occur, they are generally investigated as disparate features in the context of cancer. Such studies tend to focus either on STRs through the lens of microsatellite instability, or on protein disorder and its functional implications. Based on the findings we present here, we believe that they should rather be investigated in conjunction when one is interested in understanding their functional implications in cancer. Protein STR expansions and contractions in tumors could present a way to modulate intrinsic disorder. STR alterations that affect the state of (dis-)order in proteins may have a profound effect on their functionality and interactions. Future cancer studies should therefore be mindful of this interplay in order to arrive at a deeper understanding of the biological effects of protein STR instabilities.

## Data Availability

Publicly available datasets were analyzed in this study. This data can be found here: https://www.uniprot.org/uniprot/?query=*&fil=organism%3A%22Homo+sapiens+%28Human%29+%5B9606%5D%22+AND+reviewed%3Ayes http://current.geneontology.org/products/pages/downloads.html https://www.proteinatlas.org/humanproteome/pathology.

## Supplementary Material

**DATA SHEET 1** | Number of STR-containing proteins that correlated with patient survival for each anatomical site and correlation type (favourable/unfavourable) in the Protein Atlas Pathology Atlas.

**DATA SHEET 2** | Enriched terms for STR containing proteins from the three Gene Ontology domains (Cellular Component, Biological Process, Molecular Function), as well as enriched KEGG pathways.

**DATA SHEET 3** | Supplementary figures.
