# Supplementary material for: Beyond Microsatellite Instability: Intrinsic Disorder as a Potential Link Between Protein Short Tandem Repeats and Cancer
Source: Front Bioinform. 2021 Jun 8;1:685844. doi: 10.3389/fbinf.2021.685844 (PMC9581044; doi:10.3389/fbinf.2021.685844)
Supplement: Supplementary file 1 [file DataSheet3.PDF]

Supplementary figures accompanying:
'Beyond Microsatellite Instability: Intrinsic Disorder as a Potential Link Between Protein Short Tandem Repeats and Cancer'

By:
Max A. Verbiest, Matteo Delucchi, Tugce Bilgin Sonay & Maria Anisimova

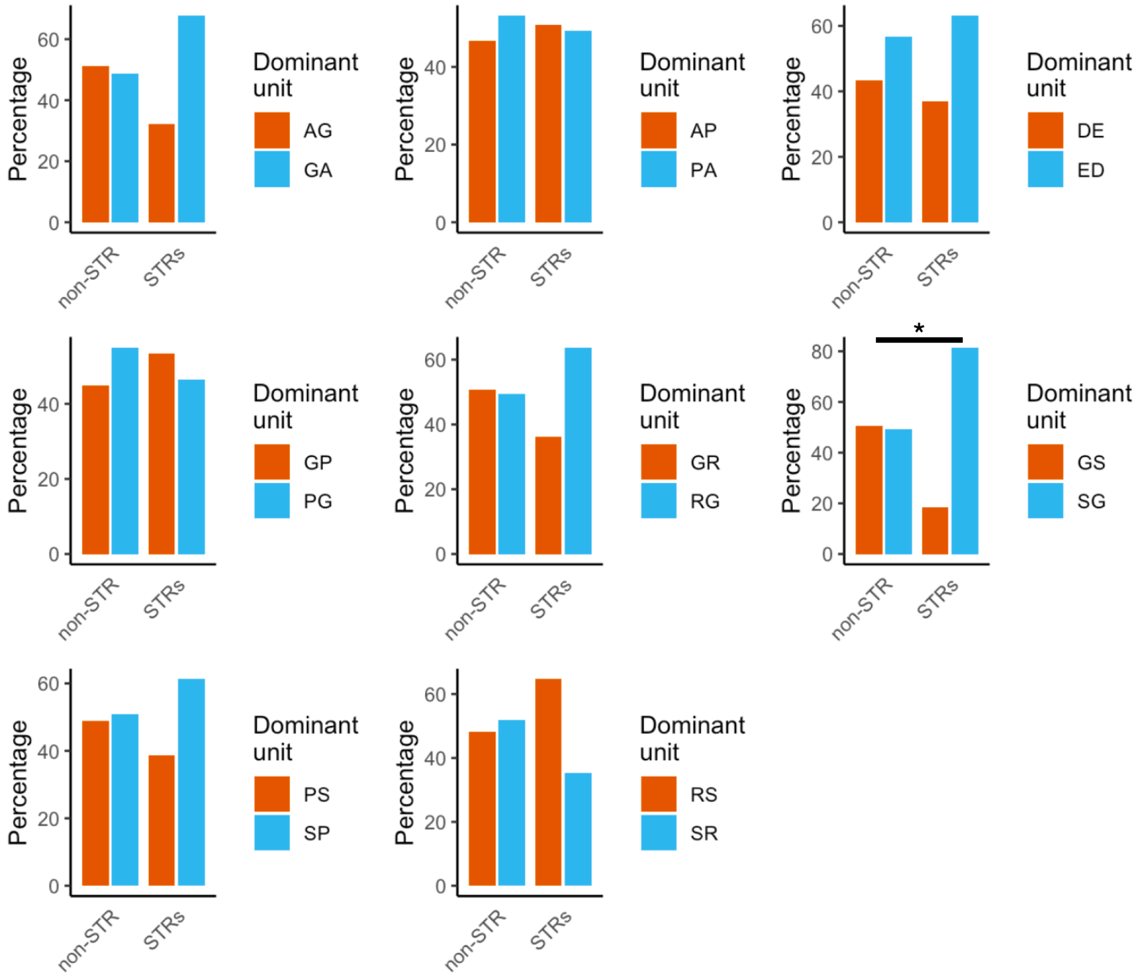

Supplementary Figure 1. Comparison of the order of amino acids in dipeptide STRs compared to non-repeating sequence. For the eight combinations of amino acids that were observed more than 30 times in STRs, the percentages of the two possible conformations are shown ('STRs'). For each amino acid combination, the order of appearance was also counted in the non-repeating sequence of all SwissProt proteins investigated in this report ('non-STRs'). Distributions that differed significantly between STRs and non-STRs according to Bonferroni-corrected Fisher's exact test are marked with an asterisk.

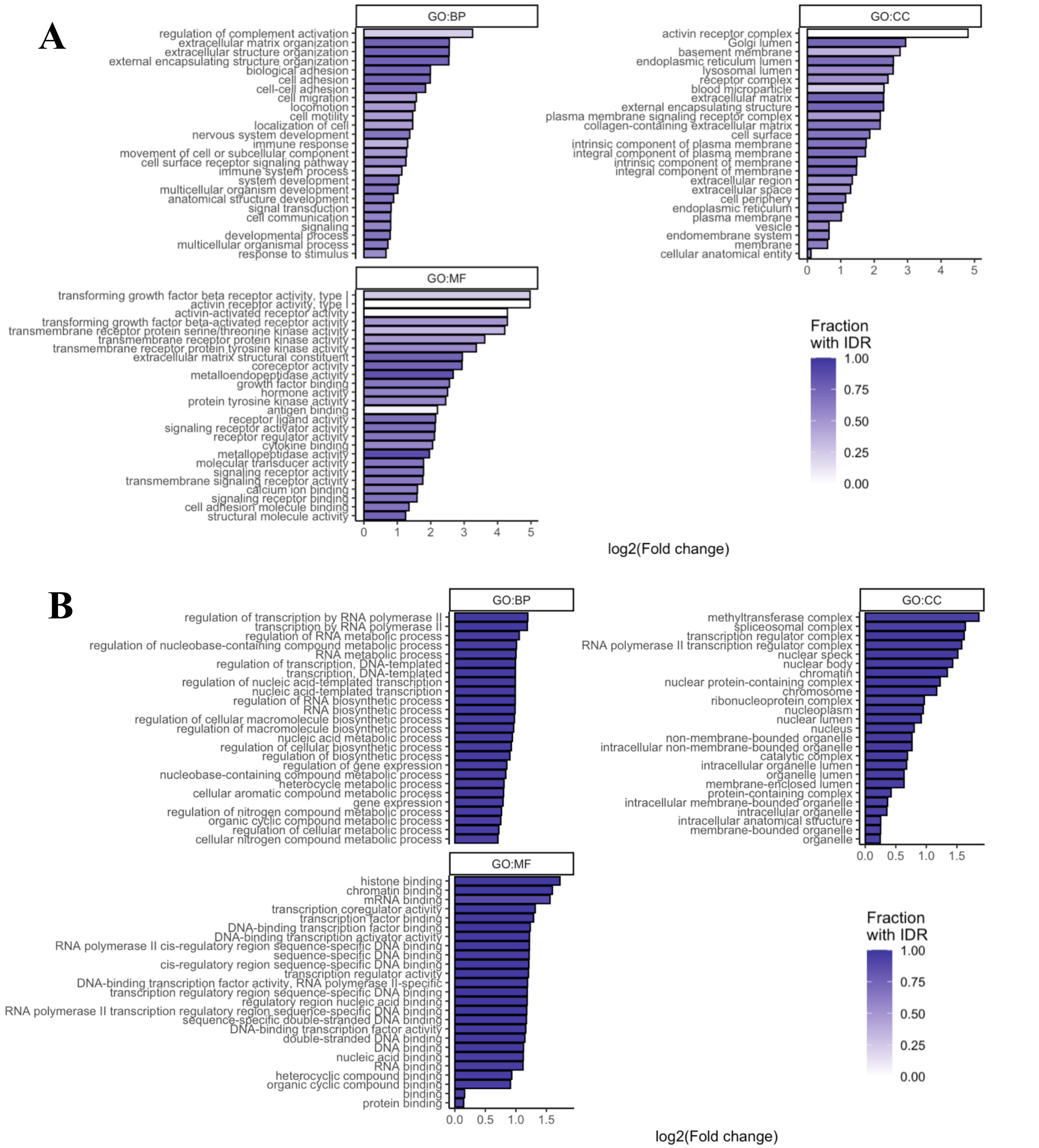

Supplementary Figure 2. g:Profiler over-representation analysis (ORA) of STR-containing proteins. log2(fold enrichment) of significantly over-represented terms for the three gene ontology domains: biological process (GO:BP), cellular component (GO:CC) and molecular function (GO:MF). **(A)** ORA for only STR-containing proteins that were annotated with a signal peptide (n=508) **(B)** ORA for only proteins that contain a disorder promoting STR and were not annotated with a signal peptide (n=1875). For each data source, the 25 over-represented terms with the lowest p-value are shown. Bars are shaded based on the fraction of proteins per term that contained an IDR.

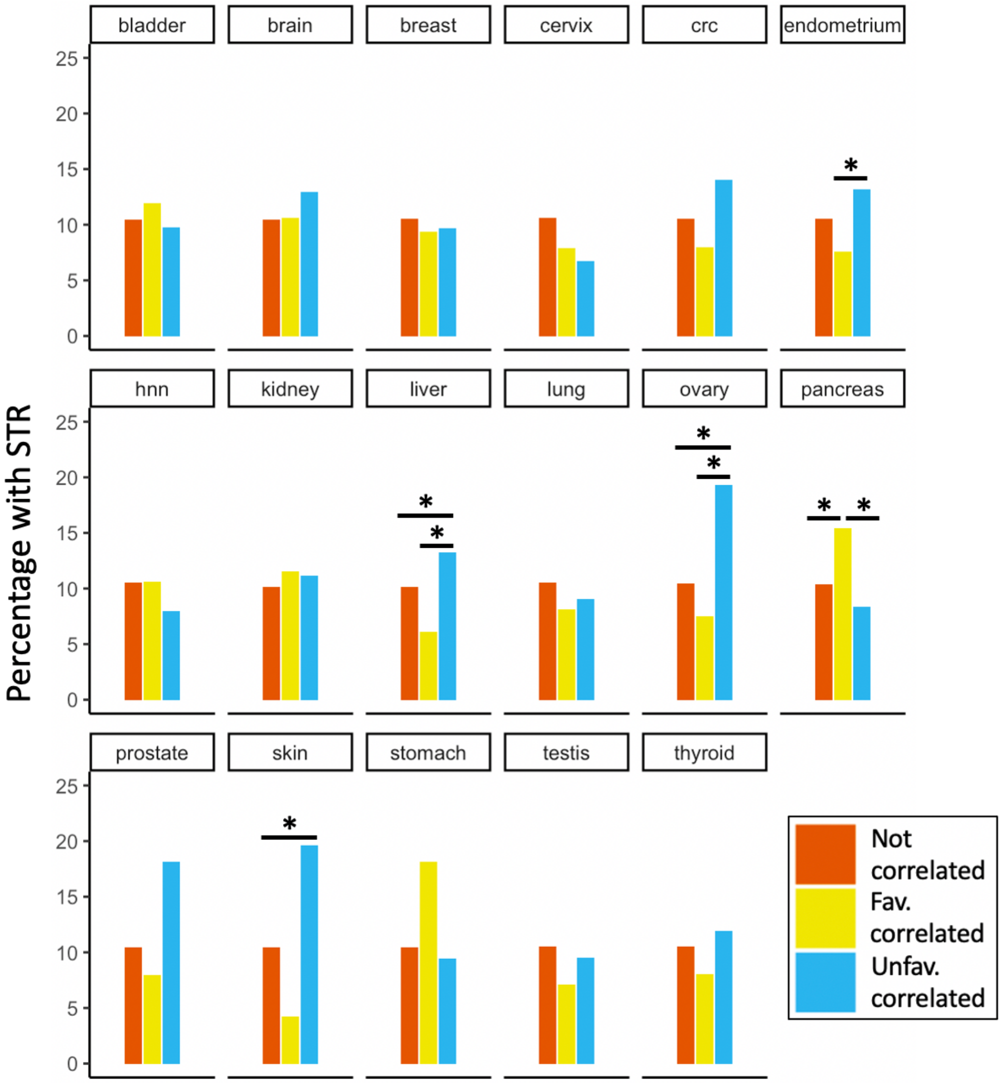

Supplementary Figure 3. Disorder promoting STR-containing proteins and their correlation to patient survival in cancer. For all 17 cancer types included in the Protein Atlas Pathology Atlas, the percentage of disorder promoting STR-containing proteins among the proteins that were either uncorrelated or correlated (un-)favourably with patient survival are shown. Significant enrichments after Benjamini-Hochberg FDR correction are marked with asterisks. Note that while percentages are shown in the plot for clarity, tests were performed on absolute numbers of proteins. Abbreviations: hnn - head and neck, crc - colorectal.

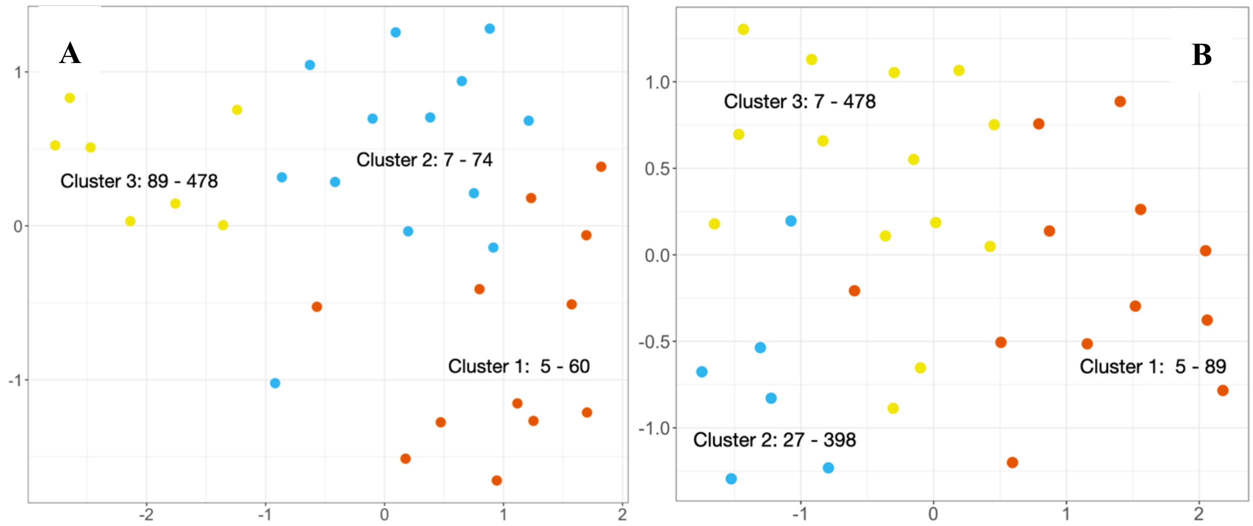

Supplementary Figure 4. Clustering biological functions of STR-containing proteins in the Protein Atlas Pathology Atlas. K-means clustering of a UMAP embedding of the number of STR-containing proteins associated to Gene Ontology terms for all PA groups. Text annotations in the graphs refer to the PA groups per cluster with the smallest and largest number of STR-containing proteins. **(A)** Analysis performed on log transformed count data **(B)** Analysis performed on count data scaled by the number of STR-containing proteins per PA group. Note: while clustering was performed on 10-dimensional embeddings, the results are visualized here using a 2-dimensional embeddings.
